# *Streptococcus cristatus*: An uncommon cause for a common hospital admission

**DOI:** 10.1016/j.idcr.2024.e02004

**Published:** 2024-05-28

**Authors:** Usamah Al-Anbagi, Aram Salehi, Godwin Justus Wilson, Fatima Noor, Ainas Osman Mohamed, Aliyaa Haji, Abdulqadir J. Nashwan, Hafsa Abdul Nasir, Bassem Al Hariri

**Affiliations:** a Medicine Department, Hazm Mebaireek General Hospital, Hamad Medical Corporation (HMC), Doha, Qatar; b Department of Laboratory Medicine and Pathology, Hamad Medical Corporation (HMC), Doha, Qatar; c Hamad Medical Corporation (HMC), Doha, Qatar; d Weill Cornell Medicine, Doha, Qatar; e College of Medicine, Qatar University, Qatar

**Keywords:** Community acquired pneumonia, Streptococcus cristatus, Ceftriaxone, Bacteremia, Infective endocarditis, Blood culture

## Abstract

Common organisms associated with community-acquired pneumonia include *Streptococcus pneumoniae*, *Haemophilus influenzae*, and *Staphylococcus aureus*. Pneumonia can rarely be caused by an organism such as *Streptococcus cristatus*, as in our case. This organism belongs to the Mitis group within the *Streptococcus genus* and typically coexists with humans in the oral cavity. We present a case of *Streptococcus cristatus* bacteremia and community acquired pneumonia in a previously healthy 40-year-old male, for whom infective endocarditis has been ruled out, and who was successfully treated with ceftriaxone. While most reported cases of *Streptococcus cristatus* involve infective endocarditis, our case is the first identified instance of community acquired pneumonia caused by *Streptococcus cristatus*. This case highlights that pneumonia with *Streptococcus cristatus*, typically considered a commensal in the oral mucosa microbiota of humans, is possible, as seen in our case. Unlike previous cases in the literature, our patient did not have infective endocarditis, which is the common presentation of this bacterium. Instead, he solely presented with pneumonia, marking the first reported case in the literature of *Streptococcus cristatus* causing pneumonia.

## Introduction

Community-acquired pneumonia (CAP) is defined as an infection of the lung tissue in an individual who contracted the infection outside of a healthcare setting. The diagnosis is confirmed through chest X-ray imaging after a patient presents with clinical symptoms of typical pneumonia, such as fever, difficulty breathing, coughing, and sputum production.

Common microorganisms associated with CAP, presenting as typical pneumonia, include *Streptococcus pneumoniae*, *Haemophilus influenzae*, *Staphylococcus aureu*s, group A streptococci, *Moraxella catarrhalis*, anaerobes, and aerobic gram-negative bacteria. Atypical pneumonia, on the other hand, manifests with a nonproductive dry cough, dyspnea, and extrapulmonary symptoms like headache, myalgia, and sore throat. It is often caused by *Legionella pneumophila*, *Mycoplasma pneumoniae*, *Chlamydophila pneumoniae*, and *Chlamydia psittaci* [1].

This case report discusses a rare instance of pneumonia caused by Group B Streptococcus, specifically *Streptococcus Cristatus* (S. cristatus). This organism belongs to the Mitis group within the *Streptococcus genus* and typically coexists with humans in the oral cavity. The organism's characteristics were first identified in 1991, initially described as "Gram-positive, catalase-negative cocci measuring approximately 1 µm in diameter and forming chains" [2].

Instances of infections caused by *S. cristatus* appear to be uncommon, with only a few reported cases in the literature that can be counted on the fingers. Our case is the first reported instance of pneumonia caused by the *S. cristatus* microorganism. (Table 1) [2].

**Table 1:** Demonstrating cases of *S. cristatus* infections [2].

Patient	Diagnosis	Infection Source and Any Coinfections	Complications	Treatment
52 y/o male, with history of epilepsy [5]	Infective Endocarditis	Aortic valve *Staphylococcus aureus*	Severe Aortic and Mitral insufficiency. Mitral perforation. Left-sided heart failure.	Failed ceftriaxone and metronidazole. Treated then with: Ampicillin, Gentamicin, Piperacillin-tazobactam, and Vancomycin
37 y/o male, previously healthy [5]	Bacteremia and Infective Endocarditis	Hematogenous spread *Streptococcus mitis*	cardiac vegetations with Severe aortic insufficiency	Ampicillin and Gentamicin. Aortic valve replacement
57 y/o male with neurofibromatosis type 1 and mitral valve prolapse [6]	Bacteremia and Infective Endocarditis	Blood	Immunocomplex glomerulonephritis	Penicillin, then Mitral Valve Repair with resection.
59 ye/o male with end-stage cryptogenic cirrhosis and aortic regurgitation	Bacteremia with possible Endocarditis	Blood	No complication	Ceftriaxone transitioning to cefpodoxime after discharge
59-year-old woman with history of glaucoma[7]	Endophthalmitis	Vitreous fluid	Left-sided bleb-related endophthalmitis	59-year-old woman with history of glaucoma[7]
66-year-old male with Myasthenia gravis, Diabetes Mellitus, Hypertension, and chronic kidney disease[8]	Vertebral discitis and osteomyelitis	Oral cavity	No complication	oral amoxicillin for an 8-week course
72-year-old patient with history of bilateral pulmonary thromboembolism secondary to deep vein thrombosis in the left leg and biological aortic valve replacement[2]	Bacteremia and Infective Endocarditis	Blood cultures + PCR 16 s on the resected valve	Splenic infarction with ischemic colitis	ceftriaxone for 6 weeks
76-year-old male with a history of aortic valve replacement and partial hepatectomy due to liver cirrhosis with hepatocarcinoma[9]	Bacteremia and probable endocarditis	Blood cultures	Small infarcts in the lower pole of the spleen and left kidney were found	Vancomycin (1 g/12 h) and cefepime (2 g/24 h) replaced by Ceftriaxone
A 66-year-old male with no prior medical history[10]	Spondylodiscitis	epidural abscess culture	No complication	V ceftriaxone (2 g bid) for 13 days then followed by oral levofloxacin (500 mg bid) for total of 6 weeks
3-year-old female with a history of mental retardation and epilepsy[5]	Transient bacteremia	Blood cultures	No complication	Amoxicillin-clavulanic acid
2 patients, no information[11]	Bacteremia and IE	Blood cultures	No information	No information
15-day-old immunocompetent male[12]	Septic arthritis of the wrist	Synovial fluid of the wrist, culture	No complication	Vancomycin for 4 weeks. Aspiration followed by arthrotomy
14-year-old female with 22q11 deletion syndrome and Tetralogy of Fallot[13]	PCR 16 s on pulmonary valve	Bacteremia and IE	Pulmonary valve replacement	Cefepime + vancomycin + gentamicin replaced after with ceftriaxone + daptomycin

## Case presentation

A 40-year-old male with no medical history was admitted to the emergency department due to a persistent fever, present for the past five days. The patient reported no complaints of cough, sore throat, hemoptysis, abdominal pain, nausea, vomiting, joint pain, dysuria, urinary frequency, rigors, night sweats, or weight loss. Additionally, there was no recent travel history or exposure to sick contacts. Otherwise, the patient was in good health.

On physical examination, the patient exhibited pyrexia with a fever of 38.2 °C, and few crackles were noted upon auscultation of the right upper lobe. Laboratory investigations revealed microcytic anemia along with an elevated C-reactive protein (CRP) count (Values in Table 2).

**Table 2:** Patient’s laboratory data:.

Parameters	On admission	3rd day	On discharge	Reference values
Total leukocytes	6.2	5.1	7.3	(6.2 ×10^3/uL)
Serum potassium K (mmol/L)	4.1	4.3	4.4	(3.5-5.3)
Serum sodium (mmol/L)	134	136	137	(133-146)
Serum calcium (mmol/L)	2.45			(2.2-2.6)
Serum urea (mmol/L)	4.6	4.2	4.5	(2.5-7.8)
Serum creatinine (umol/L)	78	61	60	(62-106)
Serum glucose (mmol/L)	5.3	5.6	5.6	(<11.1)
Serum albumin (gm/L)	37	37	33	(35–50)
Serum total protein (gm/L)	78		75	(60–80)
Lactate (mmol/L)	1.5			(0.5-2.2)
AST (IU/L)	78		50	(0-41)
ALT (IU/L)	40		68	(0-41)
Alkaline phosphatase (U/L)	110		110	(40–129)
TSH (mIU/L)	3.8			(0.3-4.2)
FT3 (pmol/L)	13.9			(11-23.3)
Serum total bilirubin (mg/dl)	6		3	(0-21)
Serum bicarbonate (mmol/L)	25.8			(22–29)

AST: Aspartate Aminotransferase. ALT: Alanine Aminotransferase. TSH: Thyroid Stimulating Hormone. FT3: Free Triiodothyronine.

**Fig. 1:**
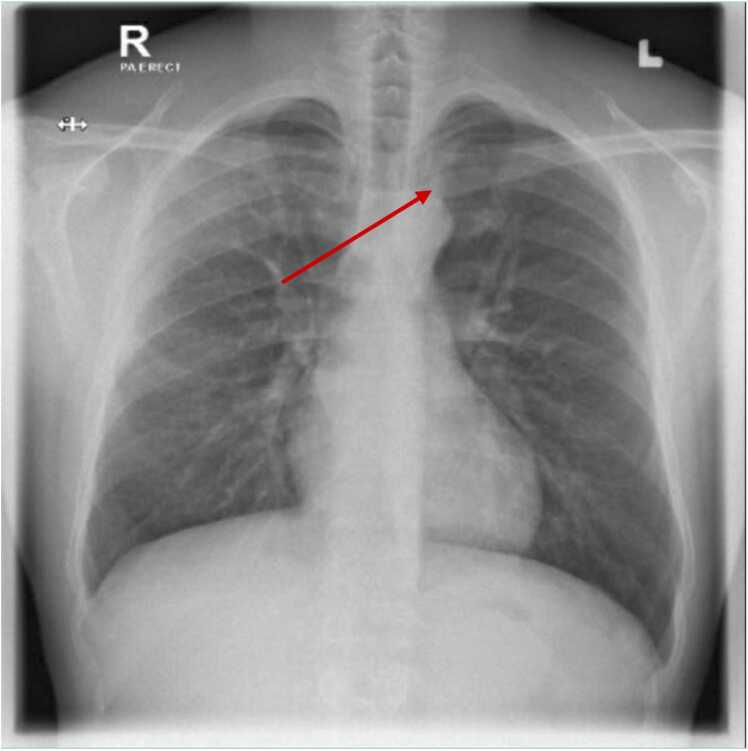
Chest X-ray demonstrating hazy opacities and inhomogeneous infiltrations in the right upper lung.

A chest radiograph revealed hazy opacities and inhomogeneous infiltrations in the right upper lung zone (Fig. 1).

**Fig. 2:**
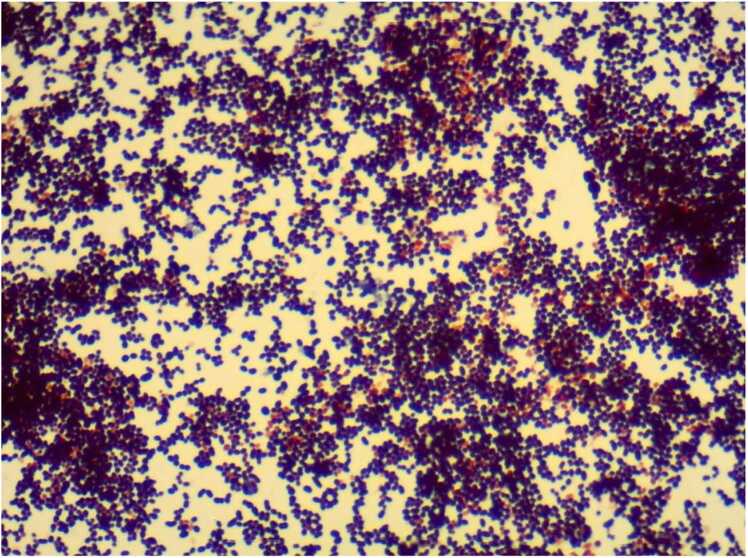
Microscopic view of Gram Stain Culture demonstrating *S. cristatus*.

The patient was admitted to the medical ward's isolation room and screened with blood and sputum cultures for infectious diseases. He was empirically treated with IV Ceftriaxone 2 g once daily and Azithromycin 500 milligram once daily following local antimicrobial guidelines while awaiting laboratory results (Table 2). After two negative Acid-Fast Bacilli (AFB) smears, the patient was removed from isolation. Subsequently, blood culture (1 out 1) results revealed *S. cristatus* growth after one day, along with the presence of polymorphonuclear leukocytes in the sputum gram stain. (Fig. 2).

The possible diagnosis of pneumonia secondary to *S. cristatus* bacteremia was established. Empiric antibiotics were discontinued, and the patient was treated with IV Ceftriaxone 2 g daily for seven days. Echocardiography was performed, along with repeated blood cultures after 72 h.

The expected outcome of the treatment plan included symptomatic relief of pyrexia and a reduction in physical examination findings, particularly the crackles in the right upper lobe of the lung. Echocardiography revealed no signs of vegetation on any heart valves.

After 72 h, repeated blood cultures showed no growth. The patient was then discharged on oral amoxicillin-clavulanate 1 g twice daily, following the recommendation of the Infectious Disease department. One month later, the patient returned for an outpatient follow-up with no signs or symptoms of the initial diagnosis.

## Discussion

S. cristatus bacteremia is a rare cause of community-acquired pneumonia (CAP) with an unknown etiology. The disease is linked to hematogenous spread through a breach in the oral mucosa lining, possibly attributed to poor oral hygiene. Additionally, the microorganism has been associated with dental caries [3]. *S. cristatus* also plays a crucial role in maintaining the oral microbiota. Studies have demonstrated its antagonizing effect on *Streptococcus mutans*, a cariogenic microorganism considered a risk factor for cancer and tumor progression [4].

Our case of community-acquired pneumonia (CAP) secondary to *S. cristatus* bacteremia presented with a low-grade fever persisting for one week without cough. Notable findings included crackles in the unilateral upper lobe of the lung, with significant radiographic evidence of hazy opacities. Given the association of *S. cristatus* with infective endocarditis, an echocardiography was performed, revealing no signs of endocarditis. The management included empiric antibiotics with IV ceftriaxone and azithromycin. Ceftriaxone was continued for seven days, followed by a course of amoxicillin-clavulanate to maintain remission. There were no additional complications throughout the treatment course, and the overall mortality associated with the infection is low [6]. Blood and sputum cultures played a crucial role in establishing the diagnosis in this patient. Treatment with antibiotics and education on the importance of oral hygiene were key factors in successful management, leading to a prosperous discharge with no recurrences of the infection.

## Conclusion

This case highlights that pneumonia with *S. cristatus*, typically considered commensal in the oral mucosa microbiota of humans, is indeed possible, as demonstrated in our case. Unlike previous cases in the literature, our patient did not present with infective endocarditis, which is the common manifestation of this bacterium. Instead, he solely exhibited pneumonia, marking the first reported case in the literature of *S. cristatus* causing this respiratory infection.

## Ethics approval and consent to participate

The article describes a case report. Therefore, no additional permission from our Ethics Committee was required.

## Consent for publication

The consent for publication was obtained from the patient.

## Funding

This study was not funded.

## CRediT authorship contribution statement

**Bassem Al Hariri:** Writing – review & editing, Writing – original draft. **Usamah Al-Anbagi:** Writing – review & editing, Writing – original draft. **Aram Salehi:** Writing – review & editing, Writing – original draft. **Godwin Justus Wilson:** Writing – review & editing, Writing – original draft. **Fatima Noor:** Writing – review & editing, Writing – original draft. **Ainas Osman Mohamed:** Writing – review & editing, Writing – original draft. **Aliyaa Haji:** Writing – review & editing, Writing – original draft. **Abdulqadir J. Nashwan:** Writing – review & editing, Writing – original draft. **Hafsa Abdul Nasir:** Writing – review & editing, Writing – original draft.

## Declaration of Competing Interest

The authors declare that they have no known competing financial interests or personal relationships that could have appeared to influence the work reported in this paper.

## Data Availability

All data generated or analyzed during this study are included in this published article.
